# Registered Clinical Trials of Ayahuasca and DMT: A Scoping Review

**DOI:** 10.1002/cpt.70311

**Published:** 2026-05-08

**Authors:** Tijana Stojanović, Kent W. Nilsson, Robert Fredriksson, Helgi B. Schiöth, Thiago C. Moulin

**Affiliations:** ^1^ Department of Immunology, Genetics and Pathology, Science for Life Laboratory Uppsala University Uppsala Sweden; ^2^ Department of Pharmaceutical Biosciences Uppsala University Uppsala Sweden; ^3^ Center for Clinical Research Uppsala University, Västmanland County Hospital Västerås Västerås Sweden; ^4^ School of Health, Care and Social Welfare, Division of Public Health Sciences Mälardalen University Västerås Sweden; ^5^ Child and Adolescent Psychiatry, Department of Medical Sciences Uppsala University Uppsala Sweden; ^6^ Functional Pharmacology and Neuroscience, Department of Surgical Sciences Uppsala University Uppsala Sweden; ^7^ Laboratory of Pharmaceutical Pharmacology Latvian Institute of Organic Synthesis Riga Latvia

## Abstract

Interest in ayahuasca and its main component, *N*,*N*‐Dimethyltryptamine (DMT), has currently moved from historical and experimental use into modern clinical development. Yet, current evidence is fragmented, and systematic mapping of trial methods and design choices remains limited. We therefore systematically examined registered interventional trials of DMT, ayahuasca, and DMT combined with harmine on ClinicalTrials.gov, identifying 26 eligible trial registers for review. We extracted and harmonized trial characteristics, participant eligibility and enrollment patterns, design features, administration routes, and registered outcomes, and linked completed registrations to associated publications. The registry landscape expanded after 2020–2021 and was dominated by early‐stage development, with most trials in phase I and more than half listed as completed at the time of extraction. Trials were primarily DMT‐only and most often sponsored by academic or hospital institutions. Eligibility criteria were conservative, emphasizing medically and psychiatrically healthy adult cohorts and extensive cardiovascular and psychiatric exclusions. Accordingly, primary outcomes prioritized acute safety and physiological monitoring, alongside structured characterization of the subjective and altered‐states profile, while disorder‐specific symptom endpoints were less commonly prioritized as primary objectives. Publications linked to included trials largely reflect this early‐stage focus, describing controlled administration, tolerability limits, route and formulation refinement, and initial mechanistic readouts. A smaller set of publications from depression‐focused trials provides preliminary evidence of potential clinical effects, supporting further controlled replication and broader disorder‐focused development. Overall, registered trials indicate an active and maturing field that has generated foundational safety and regimen knowledge, but remains constrained by a limited number of indication‐specific programs beyond depression.

**Study Highlights**

**WHAT IS THE CURRENT KNOWLEDGE ON THE TOPIC?**

Ayahuasca and *N*,*N*‐dimethyltryptamine (DMT) show mechanistic and therapeutic promise in preclinical models and early human studies, but clinical evidence remains fragmented. Trial methods vary widely, and it has been unclear how the registered clinical development landscape is evolving in terms of populations, designs, routes of administration, and outcome priorities.

**WHAT QUESTION DID THIS STUDY ADDRESS?**

What is the current landscape of registered interventional clinical trials of ayahuasca, DMT, and DMT combined with harmine, and what do registry patterns imply for methodological choices and future translational development?

**WHAT DOES THIS STUDY ADD TO OUR KNOWLEDGE?**

By systematically mapping 26 eligible ClinicalTrials.gov registrations, this scoping review shows that the field expanded sharply after 2020–2021, but remains predominantly early‐phase and safety research‐oriented. Trials are largely academic, frequently use intravenous DMT, and apply conservative eligibility criteria with extensive cardiovascular and psychiatric exclusions. Primary outcomes prioritize physiological safety monitoring and acute experiential phenotyping, whereas indication‐specific symptom outcomes are less commonly primary and are concentrated in depression. Publications related to the registered trials show that much of the current output concerns controllable dosing paradigms, tolerability limits, and formulation or route optimization, with a smaller subset reporting preliminary evidence of potential clinical effects.

**HOW MIGHT THIS CHANGE CLINICAL PHARMACOLOGY OR TRANSLATIONAL SCIENCE?**

Our findings provide a concrete evidence base for designing next‐stage trials and support a shift toward broader disorder‐focused programs. We also highlight design and reporting priorities that can reduce bias and improve interpretability. These insights should improve clinical development by guiding future trial designs to answer the questions that matter for regulation, clinical adoption, and health‐system implementation.

Ayahuasca is a psychoactive plant brew originating from South America^1^ and traditionally used in ritualistic and religious contexts.^2^ Its consumption leads to various psychological effects, such as changes in perception, intensified introspection, and heightened emotions.^2^ The brew consists of several active components, of which the most studied are *N*,*N*‐dimethyltryptamine (DMT) and harmine.^3^ DMT is a psychoactive tryptamine that primarily functions as a partial agonist for several serotonin receptors, including 5‐HT_2A_ and 5‐HT_2C_ receptors^4^; however, it can interact with a broader range of targets, including glutamate, dopamine D1, α1 ‐ and α2‐adrenergic receptors, and sigma‐1 receptors.^4^ DMT is also present endogenously within the brain at low concentrations, particularly at presynaptic terminals.^2^ Harmine is a β‐carboline that inhibits the monoamine oxidase‐A (MAO‐A), an enzyme that degrades DMT through oxidative deamination,^5^ and ultimately increases the cerebral availability of DMT.^6^

A growing body of research has examined the neurobiological effects of ayahuasca and DMT *in vitro* and *in vivo*. For example, DMT has been shown to modulate neurogenesis in adult mice by activating the main adult neurogenic niche, enhancing neural stem‐cell proliferation, promoting neuroblast migration, and increasing the generation of new neurons.^7^, ^8^ DMT also affects the hippocampus via sigma‐1 receptor signaling, leading to improved performance in spatial learning and memory tests compared with untreated controls.^8^ Similarly, harmine contributes to adult neurogenesis and neuroplasticity, and was shown to promote the migration, proliferation, and differentiation of neural stem cells.^9^

Preclinical models have also suggested the therapeutic potential of ayahuasca and DMT in psychiatric disorders. For instance, in a mouse model of alcohol use disorder, ayahuasca reduced the anxiogenic effects associated with ethanol withdrawal and modulated ethanol‐induced neuroplastic changes.^10^ Moreover, both ayahuasca and DMT have shown promising antidepressant effects in various depression models. In non‐human primates, acute ayahuasca administration led to long‐term reduction of depressive behaviors and associated phenotypes, such as feeding and body weight increase.^11^ Additionally, our group recently demonstrated that a single dose of DMT was able to revert depressive and cognitive‐deficit phenotypes induced by the chronic unpredictable mild stress (UCMS) depression model in mice.^12^ Using histological analysis, we also showed that DMT administration increased neurogenesis and led to a higher integration of new neurons in the hippocampus, reverting the stress‐induced aberrant neuronal migration.^12^

In addition to preclinical studies, several studies have been conducted in humans. Experimental investigations of DMT in humans date back to the 1950s, as a model of “experimental psychosis” through intramuscular administration.^13^ Pharmacologic characterization of DMT's psychological and physiological effects became more common in the 1990s and showed that injected DMT is rapidly metabolized, producing a fast onset and brief effects.^14^, ^15^ More recent investigations have employed neuroimaging approaches to characterize DMT's effects on human brain activity.^16^, ^17^ For instance, a multimodal fMRI‐EEG‐PET study found that DMT alters brain activation dynamics over time, particularly during the onset, peak, and offset of its effects, with the strongest effects in regions containing 5‐HT_2A_ receptors.^17^ Additionally, a case‐control fMRI study reported that long‐term ayahuasca users showed higher resilience scores than nonuser controls, together with distinct patterns of brain activity during an emotional processing task.^18^

Although our understanding of the effects of DMT and ayahuasca in humans is increasing, their ability to treat psychological disorders requires further research. Given the promising findings from preclinical and early human studies, ayahuasca and its components have recently been used in clinical trials to treat a range of conditions. Registered trials involving psychedelic compounds have already been examined in broader mapping and methodological analyses.^19^, ^20^ However, to the best of our knowledge, registered clinical trials involving ayahuasca and DMT have not yet been the subject of a focused overview. In this study, we provide a scoping review of the currently registered clinical trials employing ayahuasca or its components. We describe trial‐level features, such as objectives, trial phase, and recruitment status, participant characteristics (e.g., age, sex, sample size, and clinical population/condition), experimental design, and measured outcomes. Our analysis reveals prevailing design choices and key gaps, serving as a reference point for future trial designs.

## MATERIALS AND METHODS

### Systematic search of registered clinical trials

This work was conducted following a systematic approach to map evidence on currently registered interventional clinical trials administering DMT and ayahuasca, while identifying their characteristics, assumptions, methodologies, and knowledge gaps. Thus, we followed reporting guidelines from the PRISMA extension for scoping reviews (PRISMA‐ScR),^21^ adapted to a registry‐based evidence source. The PRISMA‐ScR checklist is provided as **File S1**. A limitation of the present review is that no prospectively registered or publicly accessible review protocol was prepared, as we considered this to be a rapidly evolving field and prioritized a timely synthesis of the available evidence. We searched ClinicalTrials.gov, a major public trial registry, to identify interventional studies investigating either ayahuasca or *N*,*N*‐dimethyltryptamine. The search was performed on July 14, 2025, using the ClinicalTrials.gov “Expert Search” interface with three separate keyword queries: “DMT,” “Ayahuasca,” and “*N*,*N*‐Dimethyltryptamine,” without applying additional filters or date restrictions. Records retrieved across queries were subsequently screened and deduplicated by NCT identifier. **Figure** S1 depicts the PRISMA flow diagram for our systematic search.

Eligibility screening was performed by manually reviewing each registry record and excluding entries that did not pertain to the target interventions. In particular, the abbreviation “DMT” frequently referred to unrelated terms (e.g., disease‐modifying therapy, dance movement therapy), and such records were excluded. Studies focused on 5‐MeO‐DMT rather than *N*,*N*‐DMT were also excluded. In addition, trials captured because of nomenclature overlap with psilocybin (e.g., 4‐phosphoryloxy‐*N*,*N*‐dimethyltryptamine or O‐phosphoryl‐4‐hydroxy‐*N*,*N*‐dimethyltryptamine) were excluded. Screening was performed independently by T.S. and T.C.M., and the resulting selections were subsequently compared. Data extraction was initially performed by T.S., and each included registry entry was subsequently verified by T.C.M. There were no disagreements during the screening phase. During data extraction, occasional uncertainties arose regarding the harmonization and wording of registry information; these were resolved through discussion between T.S. and T.C.M. until consensus was reached.

### Eligibility criteria

We included records registered on ClinicalTrials.gov that described interventional clinical studies in which ayahuasca or *N*,*N*‐DMT was investigated as an intervention, irrespective of recruitment status, population, or indication. We excluded records in which the search terms matched unrelated acronyms, trials involving 5‐MeO‐DMT (rather than *N*,*N*‐DMT), and records retrieved due to psilocybin‐related naming overlap. Observational studies were excluded from the final dataset.

### Data extraction and harmonization

For each included trial, data were extracted from ClinicalTrials.gov into a structured spreadsheet (Microsoft Excel). Initial extraction was conducted by T.S., after which T.C.M. independently reviewed the extracted information for all included registry entries. Any ambiguities in interpretation were resolved through discussion until consensus was achieved on the final classifications.

The following variables were extracted: NCT number, trial title, recruitment status, phase, conditions studied, intervention details (including dose when available), arm/group structure, study design features (e.g., allocation and masking when reported), sex eligibility, age eligibility, healthy‐volunteer eligibility, enrollment (actual for completed trials and estimated for ongoing trials), study type, key dates (start year, primary completion date, and study completion date), locations, sponsor and collaborating organizations, inclusion criteria, exclusion criteria, and registry‐listed outcome measures (primary, secondary, and other). When the “Detailed Description” field was not available, the “Brief Summary” field was used as the source for descriptive content. Dose and group information were typically obtained from the “Arms and Interventions” section and, when needed, from the descriptive text fields. Outcome measures were recorded as specified in the registry under primary, secondary, and other outcome fields, including descriptions and time frames when provided.

Several harmonization rules were applied to improve consistency across records. Trials labeled as “Early Phase I” were recorded as phase I. For trials labeled with multiple phases (e.g., phase I/phase II), only the earlier phase was recorded because registry entries do not allow determination of whether the study successfully progressed to subsequent phases. When inclusion and exclusion criteria were semantically overlapping representations of the same requirement, they were merged into a single criterion for clarity (e.g., a negative drug test requirement stated as inclusion vs. a positive drug test stated as exclusion). Sponsors were categorized as Academic/Hospital, Industry (Biotech/Pharma), or Individual/Independent investigators based on the sponsor entity listed in the registry. Importantly, one should note this approach is limited, as it may incompletely reflect the true funding structure of a trial, particularly when investigator‐initiated or academic studies receive support from commercial partners not clearly identified in the registry.

Additionally, we also harmonized study objectives into a set of predefined goal categories to enable consistent cross‐trial comparisons, as registry entries described trial aims with heterogeneous wording and varying specificity. This classification was based on the stated aims in the title, brief summary, detailed description, and, when relevant, the nature of the primary outcomes. Moreover, we observed the registry ‘Conditions’ field was used inconsistently across records, alternately describing the enrolled population's diagnosis or a broader research context, mechanistic target, or intended clinical application. We therefore harmonized condition labels to reflect the participant population actually studied. These definitions were based on the eligibility criteria and study descriptions, and involved both (i) recoding nondiagnostic or context‐style entries to ‘Healthy’ when the trial enrolled healthy volunteers (e.g., NCT06252506; NCT05559931), and (ii) refining vague diagnostic labels to more specific participant diagnoses when this was clearly indicated in the record.

Comparator categories were classified from the registry‐described control arm(s) and intervention descriptions. “Active placebo” was reserved for comparators/intervention arms explicitly described as active placebo in the registry or for low‐dose control conditions clearly intended to mimic some acute effects without constituting the main intervention. “Active comparator” was used for nonplacebo pharmacological comparators or alternative active interventions (e.g., another psychoactive drug or active treatment condition) that were not presented as placebo‐like controls. When registry wording was ambiguous, classification was based on the stated study rationale and arm descriptions, and resolved by reviewer consensus.

Primary outcome measures were extracted verbatim from the registry and then harmonized into a predefined set of outcome domains to enable cross‐trial comparison despite heterogeneous wording. When trials listed multiple primary outcomes, each outcome was classified independently, allowing a single trial to contribute to more than one domain. The mapping of verbatim registry outcomes to harmonized outcome groups is provided in **File S2** to make the categorization transparent and reproducible. Lastly, no formal critical appraisal of included registry records was undertaken, as the aim of this review was to map the characteristics of registered trials rather than to assess study quality or risk of bias.

### Synthesis of results

Extracted registry data were summarized descriptively. Categorical variables were reported as counts and percentages, while continuous or range‐based variables (e.g., age eligibility and enrollment) were visualized graphically. Harmonized trial characteristics were synthesized across figures and tables to describe temporal trends, design features, participant characteristics, intervention routes, and outcome domains. Figures were generated using Microsoft Excel, R, and Datawrapper (datawrapper.com).

### Identification of publications linked to registered trials

To contextualize registry findings with available empirical evidence, we performed a targeted, nonexhaustive search for publications associated with each included trial. This search was rerun and updated on March 23, 2026, in response to peer‐review recommendations. For each included trial, we searched PubMed and Google Scholar using both the NCT identifier and the investigator or institutional names listed in the registry field “Responsible Party.” Retrieved records were screened for matching trial‐linked outputs, based on explicit reporting of the NCT number or unambiguous correspondence in study design, intervention, population, and setting. All peer‐reviewed articles and preprints identified through this approach were summarized qualitatively in the Results section to illustrate the range of published outcomes emerging from trials in the present registry sample. This publication search was intended to support narrative interpretation of the registry landscape rather than to constitute an exhaustive literature review.

## RESULTS

### Selection of registered trials

The systematic search and selection process is summarized in **Figure** **S1**. The query “DMT” retrieved 136 registry records, of which 28 were identified as referring to *N*,*N*‐DMT after screening. The query “Ayahuasca” retrieved 9 records, 7 of which overlapped with the previously identified set and 2 of which were added. The query “*N*,*N*‐Dimethyltryptamine” retrieved 51 records, of which 50 had already been captured by the earlier searches; the remaining record was excluded because it referred to psilocybin‐related nomenclature overlap rather than an eligible *N*,*N*‐DMT study. After deduplication by NCT identifier, 31 unique records remained. Five observational studies were then excluded to restrict the final sample to interventional clinical trials, yielding 26 unique registered trials of DMT and/or ayahuasca for inclusion in the review.

### Registered interventional trials' timelines, phases, and statuses

**Figure 1:**
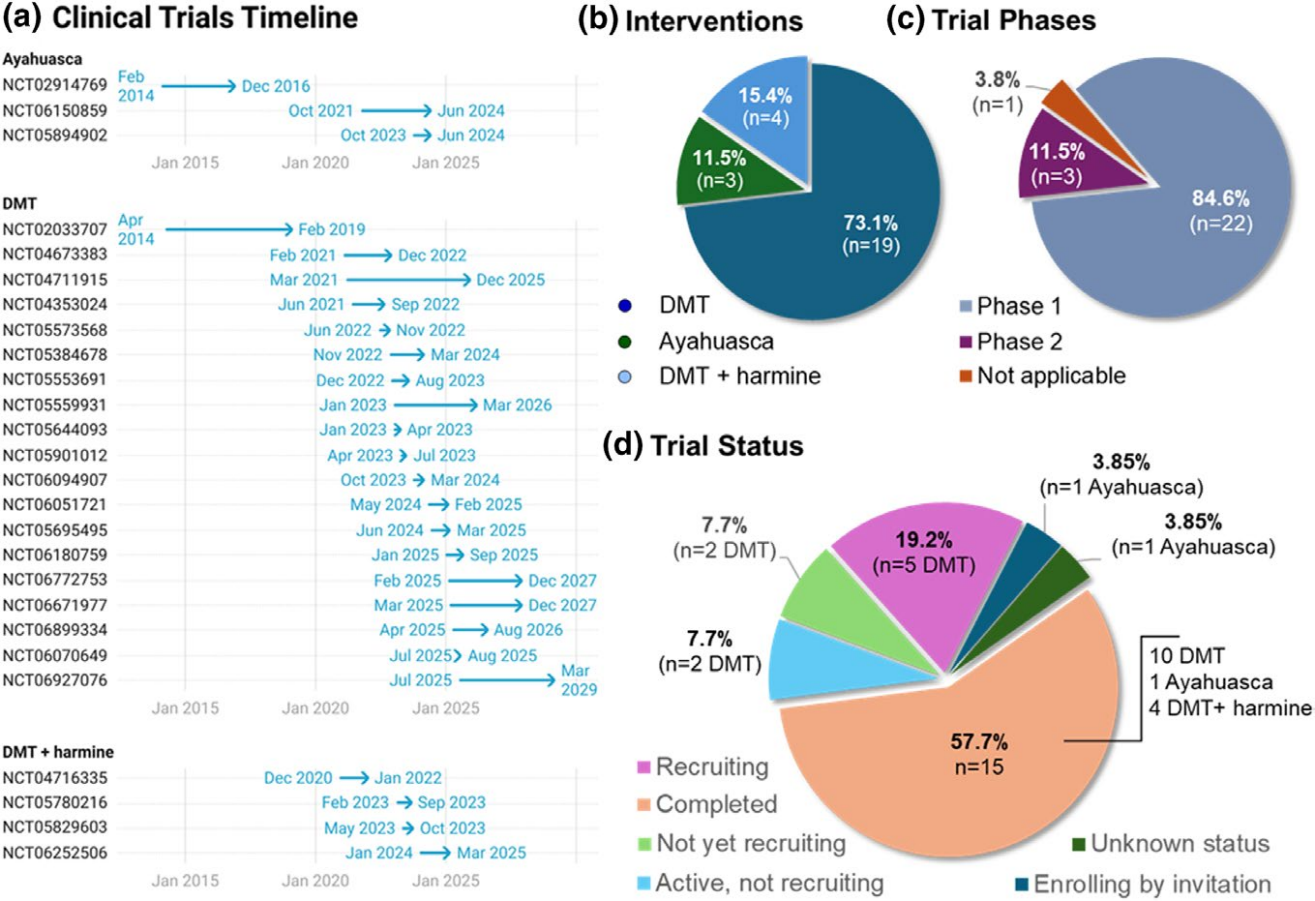
Landscape of registered interventional clinical trials of ayahuasca and DMT on ClinicalTrials.gov. (**a**) Study timelines for each included trial, grouped by intervention category (ayahuasca, DMT, and DMT + harmine). Horizontal arrows indicate the interval from the registry‐listed study start date to the listed study end date (actual or estimated at the time of extraction), with NCT identifiers shown for each record. Right panels represent distributions of (**b**) intervention category, (**c**) trial phase, and (**d**) recruitment status across the 26 included trials (percentages shown with counts). In (**c**), “Not Applicable” indicates the phase label reported in ClinicalTrials.gov for studies that do not fall within FDA‐defined trial phases. In the status panel (**d**), brackets indicate the composition of trials by intervention.

The timeline visualization highlights a sparse early period that starts in 2014, followed by a marked expansion beginning around 2020–2021, driven primarily by DMT‐only trials. Several studies initiated in 2024–2025 extend into projected completion dates beyond 2025, with the latest planned completion reaching 2029 (**Figure** **1** **a**).

Across the 26 eligible interventional trials, most registry entries evaluated DMT alone (73.1%, *n* = 19), whereas ayahuasca accounted for a smaller fraction (11.5%, *n* = 3). A further subset investigated DMT co‐administered with harmine (15.4%, *n* = 4) (**Figure** **1** **b**). The development pipeline was dominated by early‐stage studies. Phase I trials represented 84.6% of records (*n* = 22), while phase II trials comprised 11.5% (*n* = 3) (**Figure** **1** **c**).

At the time of the search, more than half of the trials were marked completed (57.7%, *n* = 15). Ongoing activity was mainly represented by recruiting trials (19.2%, *n* = 5), with additional studies listed as active, not recruiting (7.7%, *n* = 2) or not yet recruiting (7.7%, *n* = 2). Two small categories, enrolling by invitation and unknown status, each represented 3.85% (*n* = 1). Completed trials included 10 DMT‐only, 1 ayahuasca, and all 4 DMT + harmine trials, while all noncompleted trials involved DMT‐only studies except for two ayahuasca records (one enrolling by invitation and one with unknown status) (**Figure** **1** **d**). Public reporting of tabular results on ClinicalTrials.gov was also rare. Of the included trials, only NCT02914769 had results posted at the time of review, while NCT05573568 was listed as having results submitted but not yet posted. These trials are among those with associated publications, which are discussed in the following subsections.

Most trials were led by academic or hospital sponsors (65.4%, *n* = 17), with fewer sponsored by industry (Biotech/Pharma; 26.9%, *n* = 7) or individual/independent investigators (7.7%, *n* = 2) (**Figure** **S2**). Trial locations were concentrated in North America and Western Europe, with the United States and Switzerland contributing the largest share of registered study sites. Notably, Brazil was the only emerging economy with multiple registered trials, which coincides with its distinctive cultural and regulatory context surrounding *ayahuasca*, including legal allowance of use in religious practices and the brew's long‐standing ritual use among Indigenous peoples in the Amazon basin.^22^

### Participant eligibility and enrollment characteristics

**Figure 2:**
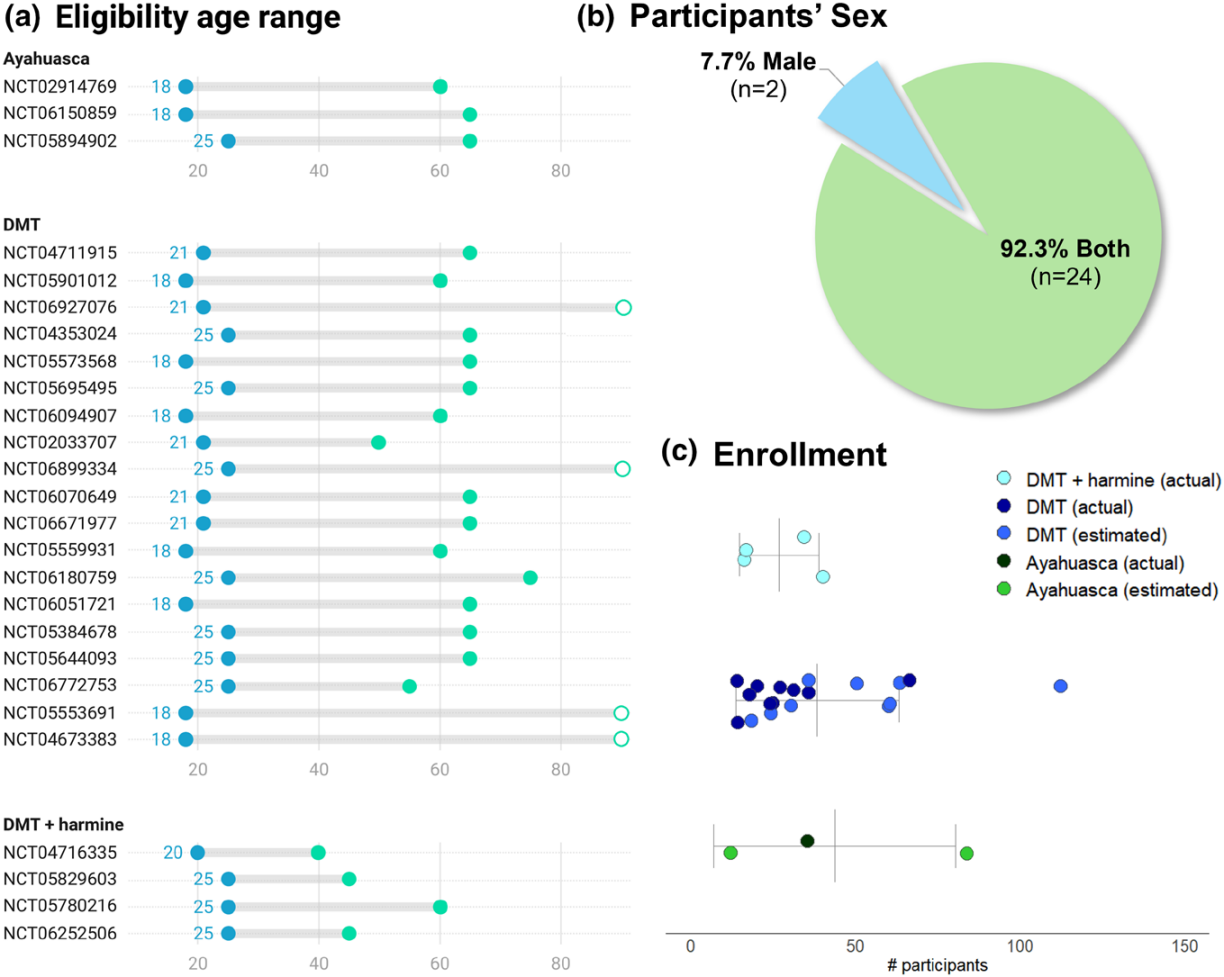
Participant eligibility criteria and enrollment across registered ayahuasca and DMT trials. (**a**) Registry‐reported eligibility age ranges for each included trial (minimum and maximum eligible age), grouped by intervention category (ayahuasca, DMT, and DMT + harmine) and labeled by NCT identifier. Upper bounds extending into advanced age or without a restrictive cap are shown as open circles at the upper end of the axis. (**b**) Distribution of sex eligibility, indicating the proportion of trials enrolling both sexes vs. male‐only eligibility. (**c**) Enrollment (actual for completed studies and estimated for ongoing studies as reported on ClinicalTrials.gov) displayed by intervention category; each point represents one trial, with vertical lines denoting the group mean and whiskers indicating ± SD.

Participant age eligibility criteria across the 26 interventional trials were largely framed around adult populations. Minimum eligible age was most commonly 18 years, although several trials set higher bounds for the minimum age (e.g., 20–25 years). Upper age limits were more variable, as many studies capped eligibility around the early older‐adult range (often ~60–70 years), while a smaller subset effectively imposed no restrictive upper bound as represented in the registry‐derived eligibility ranges (**Figure** **2** **a**). Sex eligibility was broadly inclusive. The large majority of trials enrolled both male and female participants (92.3%, *n* = 24), while two trials (7.7%) restricted eligibility to male participants only; no trials were female‐only (**Figure** **2** **b**).

Planned or actual enrollment sizes were typically modest, with most trials clustering in the small‐to‐midsize range, consistent with early‐phase development. DMT‐only trials showed the widest spread in enrollment, including one markedly larger study relative to the rest. Ayahuasca trials were few but spanned from small to comparatively larger enrollment targets, whereas DMT + harmine trials tended to fall at the smaller end of the distribution (**Figure** **2** **c**).

Across trials, eligibility criteria were broadly conservative and oriented toward minimizing medical and psychiatric risk, with the full verbatim criteria harmonized and tabulated in **Tables** **S1** and **S2**. Adults who were either psychiatrically healthy or judged cognitively capable of providing informed consent (88.5%), and many mandated peri‐session abstinence from alcohol, caffeine, nicotine, and other psychoactive substances (69.2%), often paired with requirements for a “healthy” BMI (50.0%), contraceptive use during the study (50.0%), and adequate command of the local language (42.3%) (**Table** **S1**). Exclusion criteria mirrored these priorities: pregnancy or breastfeeding (84.6%) and cardiovascular disorders (84.6%) were among the most consistently listed medical exclusions, alongside extensive psychiatric exclusions, particularly bipolar spectrum and psychotic spectrum disorders (each 80.8%), as well as restrictions related to substance‐related or addictive disorders (65.4%) and concurrent or recent psychotropic medication use (61.5%) (**Table** **S2**). Notably, despite the predominance of “healthy volunteer” frameworks, a minority of trials explicitly enrolled clinical populations (e.g., depressive disorders, grief symptoms, alcohol use disorder), indicating that the registry landscape ranges both early safety/tolerability designs and initial therapeutic evidence‐seeking studies.

### Trial objectives, target populations, and administration routes

**Figure 3:**
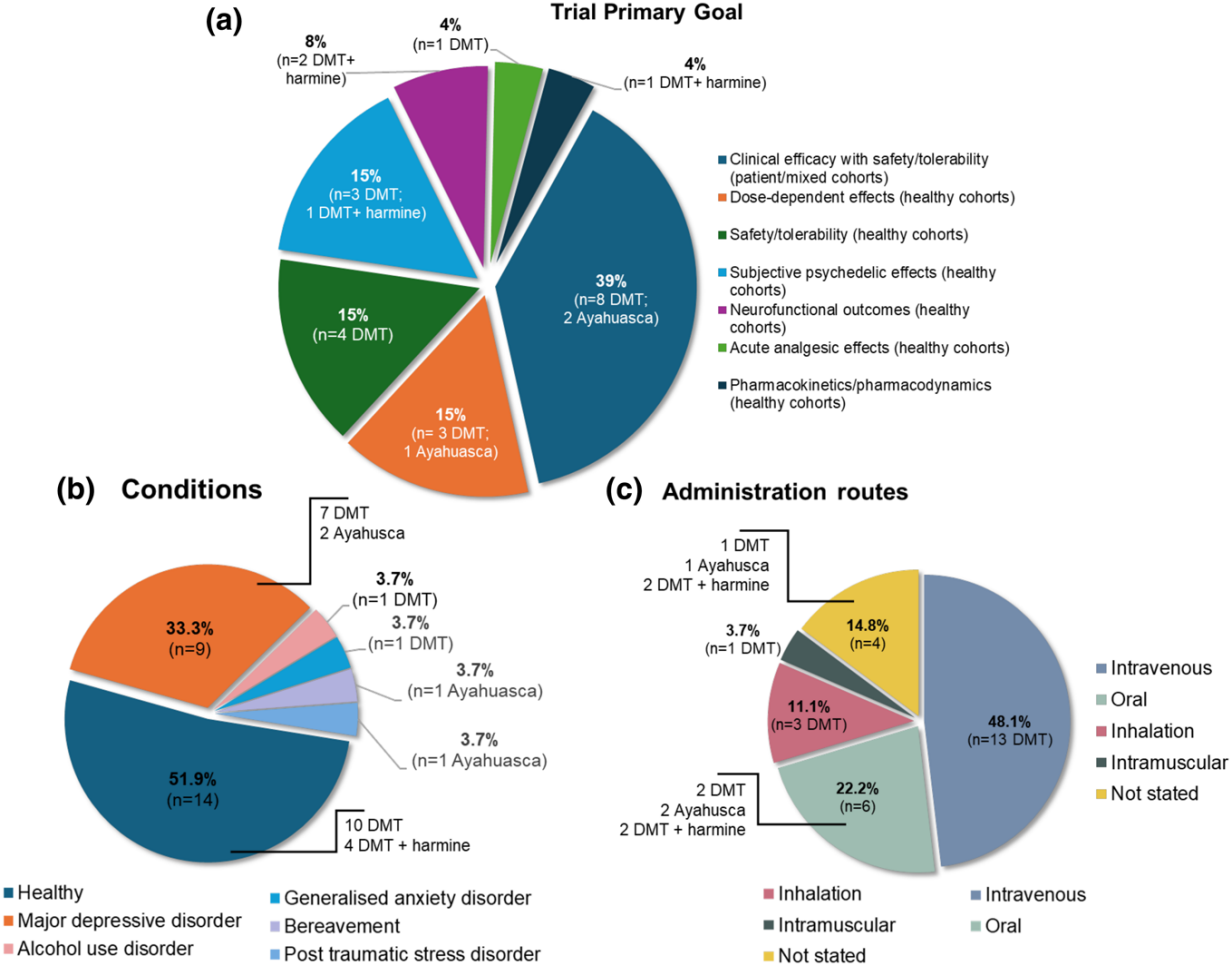
Trial objectives, participant condition categories, and routes of administration across registered ayahuasca and DMT trials. (**a**) Distribution of the primary stated goal of each registered interventional trial, categorized by the dominant focus described in the registry record. Counts are annotated with the intervention type(s) represented in each category. (**b**) Distribution of participant condition categories after harmonization of the registry “Conditions” field to reflect the enrolled population. Totals sum to 27 condition entries as one trial (NCT05894902) enrolled participants across two conditions (major depressive disorder and posttraumatic stress disorder). (**c**) Distribution of administration routes as reported in the registry. Totals sum to 27 route entries as one trial (NCT05644093) evaluated two administration routes (intravenous and intramuscular). In all panels, percentages are shown alongside counts.

The 26 registered interventional trials clustered around a small number of recurring aims (**Figure** **3** **a**). The most common primary goal was to evaluate clinical efficacy alongside safety/tolerability in patient or mixed cohorts (39%, *n* = 10). The remaining studies were largely oriented toward mechanistic and foundational clinical pharmacology in healthy cohorts, including trials focused on dose–response characterization (15%, *n* = 4), safety/tolerability in healthy participants (15%, *n* = 4), and acute subjective psychedelic effects (15%, *n* = 4). More specialized objectives were less frequent and included neurofunctional outcomes (8%, *n* = 2), as well as single trials primarily centered on acute analgesic effects (4%, *n* = 1) or pharmacokinetics/pharmacodynamics (PK/PD, 4%, *n* = 1).

Moreover, the enrolled populations were most often healthy volunteers (51.9%, *n* = 14), followed by trials enrolling participants with major depressive disorder (33.3%, *n* = 9) (**Figure** **3** **b**). The remainder of the landscape comprised single‐trial indications, including posttraumatic stress disorder, bereavement, generalized anxiety disorder, and alcohol use disorder (each 3.7%, *n* = 1).

Administration routes were similarly concentrated in a few modalities (**Figure** **3** **c**). Intravenous dosing was most common (48.1%, *n* = 13), followed by oral administration (22.2%, *n* = 6). Inhalation was used in a minority of trials (11.1%, *n* = 3), while intramuscular administration appeared rarely (3.7%, *n* = 1). Notably, a nontrivial fraction of trials did not clearly specify the administration route in the registry entry (14.8%, *n* = 4). Taken together, this profile highlights a strong preference for tightly controlled delivery paradigms, particularly intravenous dosing, alongside a smaller set of studies using more pragmatic or naturalistic routes such as oral administration.

### Core experimental design features

**Figure 4:**
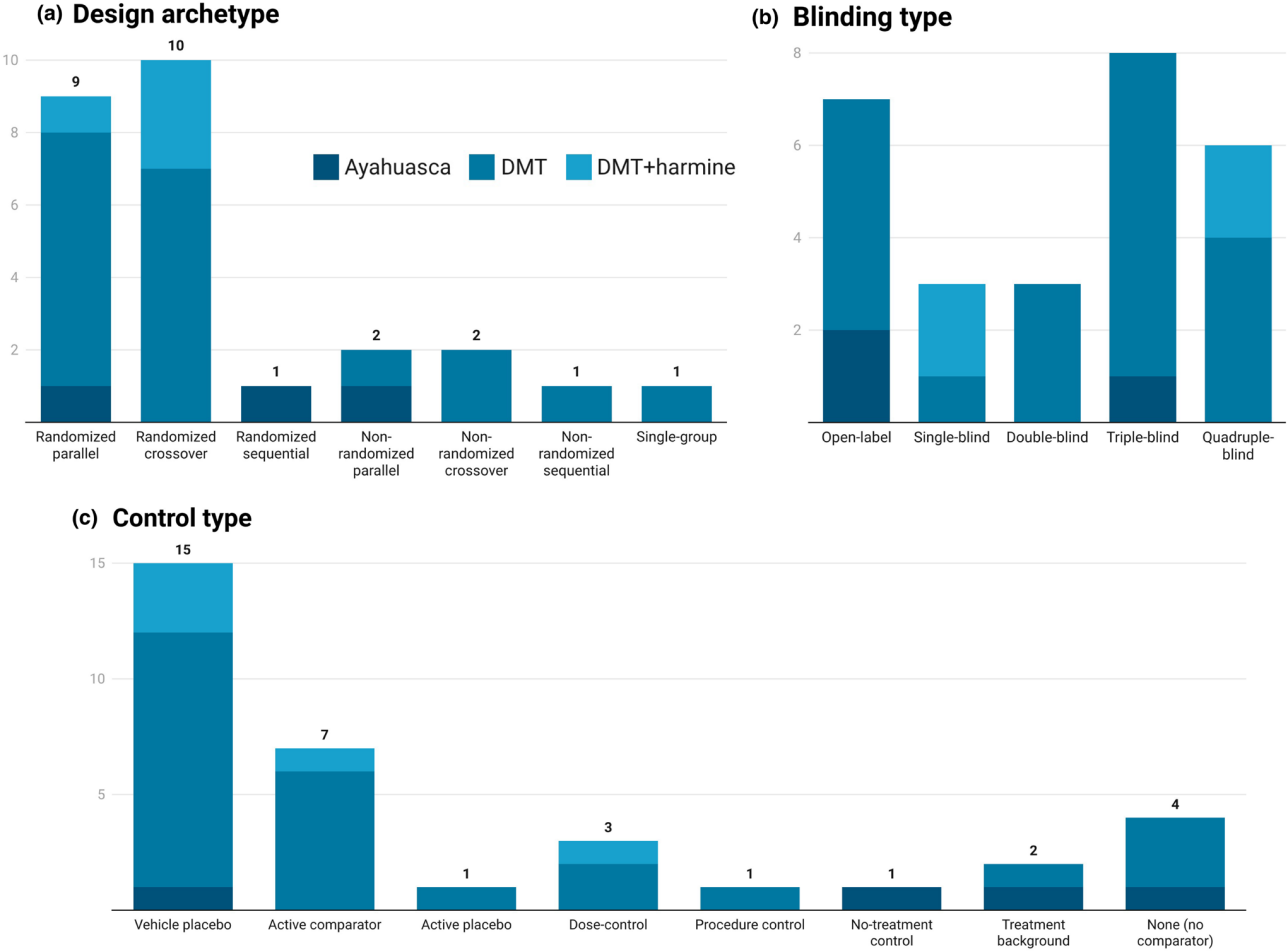
Experimental design characteristics across registered ayahuasca and DMT interventional trials. (**a**) Design archetype classification (e.g., randomized parallel, randomized crossover, sequential, and single‐group). (**b**) Masking/blinding type as reported in the registry (open‐label through quadruple‐blind). Blinding totals sum to 27 rather than 26, as one trial (NCT06927076) specified different masking schemes for different trial components (quadruple‐blind for the investigational drug and single‐blind for a sedation/procedural element) and was counted in both categories. (**c**) Control/comparator strategies reported in the registry. Control categories are not mutually exclusive because some trials used multiple control strategies; consequently, counts can exceed the total number of trials. Stacked bars indicate intervention group.

Across the 26 registered interventional trials, randomized designs clearly dominated (*n* = 20), with the remainder comprising nonrandomized or single‐group studies (*n* = 6) (**Figure** **4** **a**). Two archetypes accounted for most of the landscape, with randomized crossover trials (*n* = 10) and randomized parallel‐group trials (*n* = 9) together representing 73.1% of all studies. Less common formats included nonrandomized parallel and nonrandomized crossover studies (*n* = 2 each), and single examples of randomized sequential, nonrandomized sequential, and single‐group designs (*n* = 1 each).

Masking strategies varied from open‐label to highly blinded designs (**Figure** **4** **b**). Open‐label trials accounted for *n* = 7 studies, while the remaining trials reported some degree of blinding. Among masked studies, triple‐blind (*n* = 8) and quadruple‐blind (*n* = 6) designs were the most frequent, with fewer trials using single‐blind (*n* = 3) or double‐blind (*n* = 3) approaches. This spread is consistent with the practical constraints in psychedelic trials between rigorous masking and the difficulty of fully concealing subjective drug effects; nonetheless, the prominence of higher level masking in registry entries indicates that many groups are explicitly attempting to implement stronger blinding frameworks even in early‐phase settings.

Comparator choices were heterogeneous but showed a clear preference toward placebo‐control designs (**Figure** **4** **c**). Vehicle placebo was the most frequently listed strategy (*n* = 15), consistent with the large number of mechanistic, dose‐ranging, and safety‐oriented studies in healthy volunteers. Active comparators were also relatively common (*n* = 7), which is often used either to strengthen interpretability when an inert placebo may not adequately match experiential effects. Smaller subsets used dose control approaches (*n* = 3) or specified a treatment background (*n* = 2), while active placebo, procedure control, and no‐treatment control appeared only once each (*n* = 1). A minority of trials reported no comparator (*n* = 4), aligning with exploratory designs where the primary objective is feasibility, safety, or pharmacology rather than comparative efficacy.

Lastly, protocol transparency was limited across the registry sample. Among the included trials, only NCT06150859 was linked to a published study protocol,^23^ and only NCT05829603 explicitly described a statistical analysis plan in the registry or associated public materials.

### Primary outcome measures across registered trials

Primary outcomes, as registered and harmonized into common categories (**File S2**), showed a clear emphasis on safety/physiological monitoring and acute experiential readouts, consistent with a predominantly early‐phase trial landscape (**Table** **1**). The single most frequent primary outcome domain was vital signs (blood pressure, heart rate, and/or temperature), reported as a primary outcome in 30.8% of trials (8/26). Closely related safety endpoints were also common, including overall tolerability and safety (26.9%; 7/26), ECG‐based measures (19.2%; 5/26), and blood/urine laboratory analyses (19.2%; 5/26), alongside treatment‐emergent adverse events (15.4%; 4/26).

**Table 1:** Primary outcome measures for registered clinical trials of DMT and ayahuasca

Primary outcome measure	% Trials	Trial IDs
Change in anxiety symptoms	11.54	NCT04711915; NCT06671977; NCT06051721
Overall tolerability and safety	26.92	NCT04711915; NCT06671977; NCT06070649; NCT05559931; NCT05553691; NCT04673383; NCT05894902■
Change in behavioral outcome measures	3.85	NCT04716335●
Changes in EEG	3.85	NCT04716335●
Change in respiratory rate	7.69	NCT05901012; NCT05573568
Change in oxygen saturation	7.69	NCT05901012; NCT05573568
Changes in depressive symptoms	19.23	NCT06927076; NCT06094907; NCT06671977; NCT02914769■; NCT04673383
Altered states of consciousness profile	23.08	NCT04353024; NCT05695495; NCT06899334; NCT05384678; NCT04711915; NCT06671977
Subjective effect ratings	19.23	NCT04353024; NCT05829603●; NCT05695495; NCT05384678; NCT06671977
Incidence of treatment‐emergent adverse events	15.38	NCT05573568; NCT05829603●; NCT05644093; NCT05894902■
Pharmacokinetic parameter: *C*_max_	3.85	NCT05829603●
Pharmacokinetic parameter: AUC”	3.85	NCT05829603●
Pharmacokinetic parameter: T1/2	3.85	NCT05829603●
Blood and urine analysis	19.23	NCT05829603; NCT05644093; NCT05553691; NCT05559931; NCT05894902■
Genotyping	3.85	NCT05829603●
Subjective drug valuation and Preference	11.54	NCT02033707; NCT04711915; NCT06671977
Functional brain connectivity (rs‐fMRI)	3.85	NCT05780216●
The effects on alcohol consumption	3.85	NCT06070649
Change from baseline in cerebral metabolic rate for glucose (CMRglc)	3.85	NCT06252506●
Electrophysiological	3.85	NCT06671977
Psychotomimetic effects	3.85	NCT06671977
Pain assessment (NRS)	3.85	NCT06180759
Physical exam	11.54	NCT05644093; NCT05559931; NCT05894902■
Suicidal ideation	11.54	NCT05644093; NCT05553691; NCT05559931
Mystical Experience Questionnaire (MEQ‐30)	3.85	NCT06772753
Electrocardiogram (ECG)	19.23	NCT05553691; NCT05829603●; NCT05644093; NCT05559931; NCT05894902■
Vital signs: blood pressure, heart rate, or temperature	30.77	NCT05559931; NCT05829603●; NCT05644093; NCT05553691; NCT04711915; NCT05901012; NCT05573568; NCT05894902■
Percentage of subjects with local reactions at the injection site	3.85	NCT05559931
Occurrence of psychotic symptoms (BPRS)	3.85	NCT05559931
Occurrence of central 5‐HT toxicity	3.85	NCT05559931
Changes in grief symptom severity	3.85	NCT06150859■

Each row lists a primary outcome measure and the associated NCT IDs; percentages are calculated as the number of trials reporting the outcome divided by the total number of unique trials. Symbols appended to NCT IDs denote trial type: ■ Ayahuasca trials; ● DMT + harmine trials. Outcome categories are not mutually exclusive as some trials listed more than one primary outcome measure.

In parallel, a substantial fraction of trials prioritized acute subjective and consciousness‐related outcomes as primary endpoints, including altered states of consciousness profiles (23.1%; 6/26) and subjective effect ratings (19.2%; 5/26), reflecting a frequent focus on characterizing the phenomenology and immediate effects of the intervention (**Table** **1**). Clinical symptom outcomes were present but less dominant and concentrated in psychiatric indications: depressive symptom change appeared in 19.2% of trials (5/26) and anxiety symptom change in 11.5% (3/26). More specialized primary outcomes occurred in a small number of studies, including PK/PD parameters (each 3.85%), neurobiological readouts (e.g., rs‐fMRI connectivity, cerebral glucose metabolism, electrophysiology/EEG), behavioral tasks, pain ratings, alcohol consumption, grief symptom severity, and measures probing abuse liability or psychotomimetic effects (each 3.85%).

### Publications arising from completed and advanced trials

Several trials in the registry sample have progressed beyond registration into peer‐reviewed or preprint publications (**Table** **2**), providing an empirical window into what the field's predominantly early‐phase primary outcomes have yielded in practice. Most published reports originate from healthy‐volunteer studies designed to establish tolerability, PK/PD parameters, and controllable dosing paradigms. Across placebo‐controlled crossover experiments, intravenous DMT produced rapid‐onset psychedelic effects with short‐lived autonomic changes and rapid resolution after dosing cessation, while infusion regimens enabled more gradual, titratable exposure profiles than bolus approaches (NCT04711915,^33^ NCT04353024^34^; NCT05384678^37^). Moreover, the NCT04673383 trial investigating intravenous SPL026 (DMT fumarate) has reported phase I PK/PD and safety data in healthy participants.^30^, ^31^ More recently, the same trial also yielded a phase IIa placebo‐controlled report for MDD, in which a single intravenous dose administered with psychotherapeutic support produced a significantly greater reduction in depressive symptoms than placebo and no serious adverse events were reported.^32^ Vaporized DMT was also evaluated in healthy participants and, in a randomized double‐blind crossover comparison with active placebo, produced marked altered‐state effects while remaining well tolerated and without clinically relevant safety concerns (NCT05901012^39^). An open‐label phase I study in healthy volunteers with the same administration method showed dose‐dependent increases in subjective intensity and perceptual effects, with only mild transient cardiovascular changes (NCT05573568^36^).

**Table 2:** Publications linked to trials in the present registry sample and their principal findings

Trial (NCT)	References	Main findings
NCT02914769	Palhano‐Fontes et al.^24^	Double‐blind, randomized, placebo‐controlled trial in TRD: a single ayahuasca dose produced rapid antidepressant effects vs. placebo at days 1, 2, and 7; between‐group effect sizes increased over time; higher response rate by day 7 and a trend toward higher remission at day 7
	Zeifman et al.^25^	Secondary analysis (suicidality) from the same RCT: medium between‐group effect sizes favoring ayahuasca and large within‐ayahuasca reductions; intervention term trended toward significance (sample‐size‐limited)
	de Almeida et al.^26^	Biomarker report from the same RCT: higher serum BDNF at 48 h after ayahuasca vs. placebo; in patients receiving ayahuasca, BDNF at 48 h negatively correlated with depressive symptom severity; baseline cortisol related to BDNF patterns
NCT04716335	Dornbierer et al.^27^	Double‐blind, randomized, placebo‐controlled trial in healthy: alternative formulations/ routes (including buccal harmine + intranasal DMT) attenuated GI side effects associated with traditional oral ayahuasca (nausea/ vomiting/ diarrhea) and reduced variability in systemic exposure; overall well tolerated
	Mueller et al.^28^	Pharmacokinetics from buccal harmine + repeated intranasal DMT dosing: consistent PK profiles, acute effects resembling ayahuasca lasting ~2–3 h; harmine alone not distinguishable from placebo subjectively; conditions reported as safe and well tolerated
	Aicher et al.^29^	Secondary analysis (mindfulness/ compassion) from the same randomized, placebo‐controlled crossover trial: buccal harmine + repeated intranasal DMT increased mindfulness, self‐compassion, and compassion toward others 1 day after dosing vs. placebo, especially in high‐sensitivity participants; no lasting effects at 1 or 4 months
NCT04673383	Good et al.^30^	Phase I PK characterization from the randomized, double‐blind, placebo‐controlled dose‐escalation trial in healthy adults: IV DMT fumarate (SPL026) administered as a two‐phase infusion showed rapid attainment of peak plasma concentrations, rapid clearance with short elimination half‐life, and detailed metabolism; dose escalation was guided by safety/tolerability, with no clear relationship between peak plasma concentration and BMI/weight
	James et al.^31^	Report from the same phase I stage in psychedelic‐naïve healthy participants: SPL026 showed acceptable safety/tolerability with no serious adverse events; psychometric intensity measures increased with exposure; data used to select a target dose/regimen for subsequent patient (phase IIa) testing
	Erritzoe et al.^32^	Phase IIa randomized, placebo‐controlled MDD report from the same trial: a single 21.5‐mg IV DMT dose with psychotherapeutic support produced a significantly greater reduction in MADRS vs. placebo at 2 weeks; antidepressant effects persisted up to 3 months in the open‐label phase, with mostly mild‐to‐moderate adverse events and no serious adverse events
NCT04711915	D'Souza et al.^33^	Open‐label, fixed‐order IV DMT dose escalation (0.1 then 0.3 mg/kg) in TRD and healthy controls: generally tolerated with transient increases in BP/HR/anxiety and psychedelic/psychotomimetic effects resolving within ~20–30 min; mostly mild AEs with one self‐limited serious event; next‐day HAMD‐17 decreased after 0.3 mg/kg in TRD
NCT04353024	Vogt et al.^34^	Double‐blind, randomized, placebo‐controlled crossover IV DMT in healthy participants comparing bolus/infusion regimens: bolus produced very rapid peaks and more negative effects/anxiety than infusion; infusion enabled gradual, dose‐dependent plateaus; effects subsided rapidly after stopping; rapid PK and evidence of acute tolerance under continuous infusion
NCT06150859	Soto‐Angona et al.^35^	Open‐label, non‐randomized three‐arm clinical trial in adults with severe grief within 12 months of losing a first‐degree relative: ayahuasca‐assisted meaning reconstruction therapy was well tolerated with no serious adverse events and was associated with greater reductions in grief severity than therapy alone or no treatment, alongside improvements in prolonged grief symptoms, posttraumatic growth, and quality of life
NCT05573568	Falchi‐Carvalho et al.^36^	Open‐label, single‐ascending, fixed‐order phase I inhaled DMT trial in healthy volunteers: dose‐dependently increased subjective intensity, positive valence, and perceptual effects; produced mild, transient increases in BP/HR without clinically relevant biomarker changes; no serious adverse events, and overall was safe and well tolerated
NCT05384678	Erne et al.^37^	Double‐blind, placebo‐controlled crossover continuous IV DMT infusion in healthy participants: dose‐proportional PK and dose‐dependent subjective/autonomic effects reaching a plateau; ceiling effects for positive ratings at mid‐high dose rates and more anxiety/“anxious ego dissolution” at higher rates; self‐guided titration feasible for targeting desired intensity
NCT05780216	Meling et al.^38^	Double‐blind, placebo‐controlled DMT + harmine during a mindfulness retreat: greater mystical‐type experience, non‐dual awareness, and emotional breakthrough during acute effects; (baseline‐corrected) greater insight 1 day later; mindfulness/compassion not significantly different; at 1 month, higher ratings of personal meaning/spiritual significance/wellbeing impact vs placebo
NCT05901012	Wießner et al.^39^	Randomized, double‐blind, placebo‐controlled crossover trial in healthy participants: vaporized DMT (60 mg) was safe and well tolerated, produced transient increases in cardiovascular measures within safe limits, and induced marked subjective/altered‐state effects vs. active placebo, with mostly mild and transient adverse events
NCT05829603	Egger et al.^40^	Factorial dose‐escalation, single‐blind randomized study in healthy volunteers: transmucosal (buccal) DMT + harmine combinations (0–120 mg DMT; 0–180 mg harmine) produced reliable dose‐dependent subjective effects lasting ~4–5 h, demonstrated bidirectional pharmacokinetic interaction with harmine reducing DMT metabolism and DMT altering harmine PK, and showed an overall favorable safety/tolerability profile
	Äbelö et al.^41^	Population PK/PD modeling study based on the same trial: harmine increased DMT bioavailability and prolonged absorption/systemic exposure; subjective intensity followed a sigmoidal concentration‐effect relationship with substantial interindividual variability; simulations supported dose‐dependent potentiation by harmine and the feasibility of more precise individualized dosing strategies
NCT06094907	Falchi‐Carvalho et al.^42^	Pilot trial, open‐label, fixed‐order, dose‐escalation vaporized DMT in TRD: significant MADRS/PHQ‐9 reductions from day 1 through 1 month; high response/ remission at day 7 with partial maintenance at 1 month (small‐sample pilot)
	Falchi‐Carvalho et al.^43^	Phase 2a results of the same trial: safe/well tolerated with manageable acute effects and no serious adverse events; large MADRS reduction by day 7 with high response/remission rates and reported persistence up to 3 months; suicidal ideation decreased post‐dosing
NCT06252506	Egger et al.^44^	Single‐blind, placebo‐controlled crossover FDG‐PET in healthy males: global brain glucose metabolism increased (~12%) under DMT + harmine vs. placebo with widespread cortical/network effects; exploratory correlation between global metabolism and harmine exposure (AUC)

Listing of peer‐reviewed articles (and one preprint) identified for trials included in the present review, matched by ClinicalTrials.gov identifier (NCT number). Main findings are summarized at a high level to reflect the primary conclusions reported in each publication.

AEs, adverse events; AUC, area under the concentration‐time curve; BDNF, brain‐derived neurotrophic factor; BP, blood pressure; FDG‐PET, [^18^F]fluorodeoxyglucose positron emission tomography; GI, gastrointestinal; HAMD‐17, 17‐item Hamilton Depression Rating Scale; HR, heart rate; IV, intravenous; MADRS, Montgomery‐Åsberg Depression Rating Scale; MDD, major depressive disorder; PK/PD, pharmacokinetics/pharmacodynamics; PHQ‐9, Patient Health Questionnaire‐9; TRD, treatment‐resistant depression.

A second cluster of publications focuses on ayahuasca‐analog formulations combining DMT with harmine, with an emphasis on improving clinical usability through alternative routes of administration. Reports from NCT04716335 indicate that buccal harmine combined with intranasal DMT can produce consistent PK profiles and ayahuasca‐like acute effects while attenuating common gastrointestinal intolerabilities seen with traditional oral preparations; in this randomized placebo‐controlled setting, drug conditions were described as safe and well tolerated.^27^, ^28^ A secondary analysis from the same trial further suggested short‐term increases in mindfulness and compassion, although these effects were not sustained at longer follow‐up.^29^ Similarly, the mindfulness‐focused NCT05780216 trial showed that, when compared with meditation with a placebo, meditation combined with DMT and harmine induced greater levels of self‐attributed subjective experiences.^38^ Mechanistic neuroimaging has also begun to appear in this same formulation space, including a placebo‐controlled crossover FDG‐PET study reporting global increases in brain glucose metabolism under DMT + harmine relative to placebo^44^ (NCT06252506). Lastly, the NCT05829603 study and its subsequent PK/PD modeling analysis showed that transmucosal DMT + harmine combinations produced reliable dose‐dependent subjective effects, demonstrated bidirectional pharmacokinetic interaction between the two compounds, and supported the feasibility of more individualized dosing strategies through harmine‐mediated potentiation of DMT exposure.^40^, ^41^

Importantly, although fewer in number, publications from trials in this registry sample focused on psychiatric indications provide preliminary evidence of potential clinical effects. For instance, a double‐blind placebo‐controlled randomized trial of ayahuasca in treatment‐resistant depression reported significant antidepressant effects relative to placebo across days 1–7 with increasing between‐group effect sizes by day 7, alongside secondary reports on suicidality and serum BDNF modulation within the same trial framework^24^, ^25^, ^26^ (NCT02914769). More recently, open‐label reports of vaporized DMT in treatment‐resistant depression described rapid reductions in depressive symptoms with high response/remission proportions and persistence of benefit up to months in some participants, while remaining well tolerated under clinical monitoring^42^, ^43^ (NCT06094907). Beyond depression, one open‐label nonrandomized three‐arm clinical trial in individuals with severe grief suggested that ayahuasca‐assisted meaning reconstruction therapy may reduce grief severity more than psychotherapy alone or no treatment, while also improving prolonged grief symptoms, posttraumatic growth, and quality of life without serious adverse events (NCT06150859).^35^

Most completed clinical trials within our review could be linked to publications; however, some have not yet yielded publicly available trial results in either the peer‐reviewed literature or the ClinicalTrials.gov results database. For instance, the phase I placebo‐controlled NCT05695495, completed in March 2025, was designed to establish an intravenous DMT bolus dose–response relationship in healthy participants by relating multiple bolus to subjective and autonomic effects. Although data from this study appear to have contributed to a later psychometric revalidation of the ASC questionnaire,^45^ that publication did not report the trial's DMT findings themselves, and no dedicated publication of the registered study outcomes was identified. Similarly, NCT02033707, a phase I study completed in February 2019, was designed to characterize the effects of a broad range of hallucinogenic and other psychoactive compounds on mood and performance. Although at least one publication appears to have emerged from this broader program, the identified report focused specifically on dextromethorphan and psilocybin rather than on any DMT‐related findings from the registered protocol.^46^ Lastly, two completed phase I studies sponsored by the same company, Small Pharma Ltd, had no corresponding publications or posted tabular results. NCT05644093 evaluated intramuscular and intravenous SPL026 (DMT fumarate) in healthy participants and was completed in April 2023, whereas NCT05553691 assessed intravenous SPL026 alone or alongside SSRIs in patients with major depressive disorder and was completed in August 2023.

## DISCUSSION

In this scoping review, we systematically searched for registered interventional clinical trials of DMT, ayahuasca, and DMT co‐administered with harmine, and identified 26 eligible studies. We then characterized the landscape across timelines, intervention categories, phases/recruitment statuses, participant eligibility and enrollment patterns, trial design features, and primary outcome priorities. We also linked completed registrations to published reports when available. Overall, the registry evidence depicts an expanding but still predominantly early‐phase field, with a strong emphasis on safety/tolerability and pharmacological characterization alongside a smaller, albeit promising, set of disorder‐focused studies. More broadly, this pattern is consistent with previous mapping efforts across psychedelic research, which have likewise described a literature dominated by early‐phase studies, relatively small samples, short follow‐up periods, and methodological heterogeneity. Below, we discuss the main patterns emerging from this landscape and their relevance to ongoing methodological and regulatory debates.

### Registered trial characteristics and participant eligibility profiles

Across the registry sample, trial timing, phase, and recruitment status depict an active but still predominantly early‐phase field. Registrations were sparse before 2020, then increased markedly from 2020 to 2021 onward, driven mainly by DMT‐only programs, with several newer studies extending into projected completion dates beyond 2025. Most registered trials were phase I (84.6%); relatively few were phase II (11.5%); and none were phase III–IV. At the time of extraction, more than half were completed (57.7%), with the remainder distributed across recruiting and other active statuses. Overall, this pattern indicates a field that is expanding and operationally active, but still focused mainly on early development rather than confirmatory efficacy testing.

Participant eligibility and enrollment patterns are consistent with this profile. Most trials enrolled adult participants, with upper age limits commonly clustered around ~60–70 years and only a minority allowing older ages without a clear cap. Sex eligibility was broadly inclusive (92.3% enrolling both sexes), whereas male‐only enrollment was uncommon (7.7%). Enrollment targets were generally small to mid‐sized, with greater dispersion among DMT‐only trials and smaller samples among DMT + harmine studies. Most trials were also sponsored by academic or hospital institutions (65.4%) rather than private organizations.

Finally, the eligibility criteria themselves support a conservative, risk‐minimization approach. Most trials imposed peri‐session restrictions on alcohol/caffeine/nicotine and other psychoactive exposures (69.2%), while exclusion criteria frequently targeted pregnancy/breastfeeding and cardiovascular disorders (each 84.6%), bipolar‐ and psychotic spectrum disorders (each 80.8%), substance‐related disorders (65.4%), and concurrent/recent psychotropic medication use (61.5%). These choices strengthen internal validity by reducing heterogeneity and foreseeable risk, and by facilitating clearer attribution of acute physiological and subjective effects. However, they also constrain external validity because the populations represented in the current registry landscape are narrower and healthier than many real‐world psychiatric populations likely to be considered for these interventions in practice. As a result, the present trial landscape is informative for early pharmacology and feasibility, but less informative about how these interventions may perform in broader and more clinically complex populations.

### Trial design, controls, blinding, and evidentiary standards

In our registry sample, placebo‐controlled designs were common, with vehicle placebo used in 57.7% of trials, active comparators in 26.9%, and active placebos in only 3.8%. Open‐label designs accounted for 26.9% of studies, whereas the remainder reported some form of masking, often at higher nominal levels such as triple‐ or quadruple‐blind. Moreover, the broader design profile also strongly reflects an early‐phase landscape. Most trials were small, averaging less than 50 participants, mostly conducted in healthy volunteers (51.9%), and most prioritized dose‐finding, PK/PD characterization, route optimization, and acute safety/tolerability rather than longer term or confirmatory clinical objectives.

These features are useful to interpret in light of recent FDA‐ and EMA‐oriented methodological discussions on psychedelic drug development.^19^ For instance, regarding trial design, the current landscape appears reasonably aligned with early‐stage expectations. Many studies use randomized crossover or parallel‐group structures and focus on defining dose–response relationships, characterizing acute pharmacology, and quantifying interindividual variability in exposure and tolerability. This is broadly consistent with the type of foundational evidence expected before later‐stage therapeutic claims are pursued.^19^ However, most studies remain short in follow‐up, highly selective in eligibility, and concentrated in healthy volunteers rather than in target patient populations, thus falling short of expectations for more confirmatory development.

With respect to control groups, the predominance of inert placebo broadly resembles patterns observed in the wider psychedelic randomized trial literature, in which inactive controls remain common despite concerns that conspicuous psychoactive effects may compromise blinding and inflate expectancy‐related differences on subjective outcomes.^47^ In that sense, the present registry landscape only partially aligns with current methodological expectations. Inert placebo may be acceptable for some early pharmacology and safety questions, but it is less well suited to trials in which the main challenge is to separate drug effects from expectancy‐driven differences.^48^ The rarity of active placebo in registered DMT/ayahuasca trials is therefore notable, as active placebos are one of the more direct tools available to reduce differential expectancy while preserving trial feasibility.^47^, ^49^

Blinding raises a related concern. Although most registered trials reported some degree of masking, systematic evidence indicates that masking success in psychedelic studies is frequently poor when formally evaluated, and that trials often omit standardized assessment of blind integrity altogether.^47^, ^48^ This is especially meaningful when outcomes depend on subjective symptom scales, acute experiential ratings, or self‐attributed psychological changes. Thus, while the registry entries suggest a clear intention to blind, more attention should be given to active measurements of blind integrity. In practice, many of the identified trials remain vulnerable to functional unblinding, which makes causal attribution to the pharmacology more difficult even when clinical improvement is genuine.^48^

By contrast, the identified trials align more clearly with expectations regarding safety monitoring. Safety and tolerability endpoints were highly prominent across the sample, and many studies incorporated intensive acute monitoring, including vital signs, ECG, laboratory parameters, and adverse events. This is consistent with the current emphasis on identifying and managing acute anxiety, cardiovascular effects, drug–drug interactions, and interindividual variability in exposure.^19^ From this perspective, one of the clearest strengths of the current DMT and ayahuasca trial landscape is the amount of attention already devoted to acute safety characterization and controlled administration conditions.

The weakest alignment is with confirmatory evidence standards. Although some patient‐focused studies have now emerged, the field still contains few registered trials that approximate the scale, follow‐up duration, patient representativeness, and methodological robustness typically expected for stronger efficacy claims. Most studies are not designed to evaluate maintenance of benefit over extended periods, symptom recurrence, or the extent to which observed effects can be disentangled from accompanying psychological support. These patterns suggest that the field has progressed substantially in early clinical pharmacology and acute safety assessment, but remains less advanced in generating the kind of evidence needed for more robust clinical and regulatory conclusions.

### Registered outcomes and published clinical evidence

The pattern of registered primary outcomes suggests that the field has so far prioritized acute safety, tolerability, and controllable administration over later‐stage therapeutic testing. Most trials were designed around intensive monitoring of vital signs, ECG, laboratory parameters, adverse events, and acute subjective effects. In contrast, disorder‐relevant symptom outcomes were less often prioritized as primary endpoints, suggesting that the field's main near‐term objective has been to establish whether DMT‐ and ayahuasca‐based interventions can be delivered in a predictable, monitorable, and pharmacologically tractable way before broader expansion into indication‐specific efficacy testing.

The published evidence is broadly positive for these early‐stage objectives. Across administration routes, studies employing DMT alone or in combination with harmine consistently show rapid‐onset psychedelic effects, short‐lived autonomic changes, and overall acceptable tolerability under controlled conditions.^27^, ^28^, ^29^, ^30^, ^31^, ^32^, ^33^, ^34^, ^36^, ^37^, ^38^, ^39^, ^40^, ^41^, ^44^ Route and regimen clearly influence intensity, duration, and adverse‐effect burden, and several studies now provide converging evidence that these factors can be actively shaped through infusion strategies or alternative formulations.^27^, ^28^, ^33^, ^34^, ^37^, ^41^, ^44^ These outcomes indicate that a reasonably robust early clinical pharmacology framework has been developed for DMT‐based interventions. Although rare adverse events and longer term risks still require continued attention, the published record now provides fairly consistent support for the safety of these compounds when administered in carefully screened and monitored research settings.

The more clinically relevant question is whether these interventions produce therapeutic effects in psychiatric populations. The available studies in treatment‐resistant depression suggest rapid symptom reduction after both ayahuasca and DMT‐based interventions, with additional reports of reductions in suicidality and improvements in biomarkers such as BDNF.^24^, ^25^, ^26^, ^32^, ^42^, ^43^ Beyond depression, an open‐label trial in severe grief also suggested improvements in grief‐related outcomes and quality of life.^35^ These findings are promising, but more evidence is needed to support strong conclusions about comparative efficacy, durability, optimal target populations, or the extent to which observed benefits can be separated from expectancy, contextual influences, or accompanying psychological support. In that sense, the field has clearly advanced beyond pure safety/pharmacology work, but disorder‐focused development is still at an early stage.

A particularly promising aspect of DMT‐based interventions is that they combine features that are relatively unusual within psychedelic research, including rapid onset, short duration, flexible delivery options, and an increasingly well‐defined pharmacological profile. These features could be advantageous in settings where long‐session psychedelic treatments are difficult to implement, too resource‐intensive, or less scalable. Moreover, formulations combining DMT with harmine and alternative non‐oral routes suggest that the field is actively developing more clinically adaptable approaches. Whether these practical advantages will translate into meaningful clinical gains remains uncertain, but they do provide a clear rationale for continued development and more rigorous patient‐focused trials.

### Limitations

Importantly, this review has some limitations that should be considered. First, it relies exclusively on ClinicalTrials.gov, and registry entries are often incomplete or less detailed than full study protocols. In addition, some variables required harmonization and interpretation, potentially leading to misclassification, including for sponsor type, comparator categories, and study goals. Although these procedures were performed systematically and verified across reviewers, some degree of imprecision is unavoidable in a registry‐based analysis. Finally, the linked‐publication search was intended to contextualize the registry landscape rather than to serve as an exhaustive systematic review of the published literature.

### Perspectives on future research

#### Translational mechanistic gap

An unresolved question emerging from both the preclinical and clinical registry literature concerns dose–response relationships and their relevance for neurogenesis‐ or neuroplasticity‐related mechanisms. Preclinical studies have shown that DMT and harmine can modulate adult neurogenesis‐related processes,^7^ but the relevance of those findings to human dosing paradigms remains uncertain. This issue is not directly resolved by the present registry landscape, yet it remains relevant because a meaningful portion of ongoing clinical development is centered precisely on dose‐ranging, route optimization, and the relationship between acute experiential intensity and tolerability.

From a translational standpoint, one important question is whether putative neuroplastic or neurogenesis‐related effects, if they prove relevant in humans, are tightly linked to intense subjective experiences or could also occur at lower exposure levels. At present, this remains speculative, and the current trial landscape provides little direct evidence on the issue. Relatively few studies include explicit dose‐ranging objectives linked to downstream biological markers, and none directly assess neurogenesis‐related outcomes in humans. Accordingly, future studies incorporating multimodal proxy measures of plasticity, including neuroimaging, electrophysiology, and biomarkers such as BDNF, could help reduce this knowledge gap and contribute to future mechanistically informed treatment choices.

#### Set, setting, and implementation considerations

Beyond pharmacology and dosing, future clinical implementation will also depend on how contextual factors, such as set and setting, are integrated into treatment protocols. The current registry landscape provides limited detail on psychological preparation, environmental context, therapist involvement, or post‐session integration, yet these factors are widely recognized as relevant to both acute experiences and longer term outcomes. This is not only a clinical issue but also a regulatory one, because when psychedelic interventions are delivered together with substantial psychological support, it becomes more difficult to determine how much of the observed effect is attributable to the drug itself and how much to the therapeutic context.^19^ Future studies that specify these contextual components more clearly, and when feasible, evaluate them more explicitly, would improve both mechanistic interpretability and applied relevance.

#### Age, vulnerability, and implications for young adult populations

Another dimension that warrants explicit consideration is participant age and its intersection with both neurobiological plasticity and clinical need. Neurogenesis and broader neuroplastic processes are known to be most robust during adolescence and young adulthood, with a gradual decline across adulthood.^50^, ^51^ Coincidentally, this same developmental window, ~ up to 25–30 years of age, is also characterized by a high incidence of anxiety disorders, depressive disorders, and the initiation of substance use and addictive behaviors.^52^, ^53^ From a public health perspective, this convergence raises the possibility that interventions targeting neuroplastic mechanisms during this period may yield particularly strong or enduring effects.

Despite this theoretical alignment, most registered trials impose conservative age limits and preferentially recruit mature adults, often excluding younger individuals who may stand to benefit the most from early intervention. This caution is understandable given ethical considerations and the limited safety data in younger populations. However, it also highlights a tension between developmental neurobiology and current clinical trial practice. If ayahuasca‐ or DMT‐based interventions are shown to meaningfully reduce anxiety, depressive symptoms, or maladaptive coping behaviors, they may ultimately represent a valuable treatment option for young adults, a group in which conventional treatments are often insufficient and where long‐term disease trajectories are still questionable. Carefully staged research programs, beginning with older cohorts and gradually extending into younger adult populations under stringent safety oversight, may therefore be a rational future direction.

## FUNDING

The authors received no funding relevant to this publication.

## CONFLICT OF INTEREST

The authors declared no competing interests for this work.

## AUTHOR CONTRIBUTIONS

T.S., K.W.N., R.F., H.B.S., and T.C.M. wrote the manuscript; H.B.S and T.C.M. designed the research; T.S. and T.C.M. performed the research; T.S. and T.C.M. analyzed the data.

## Supporting information


Figure S1.

Figure S2.

Table S1.

Table S2.

File S1.

File S2.

File S3.

## Data Availability

The dataset containing all extracted variables and derived classifications generated in this study is provided in **File S3**. The full extracted dataset and derived variables used for figure generation are provided in **File S3** and are also publicly available via Synapse (https://doi.org/10.7303/syn74297841.1).
