# Supplementary material for: Registered Clinical Trials of Ayahuasca and DMT: A Scoping Review
Source: Clin Pharmacol Ther. 2026 May 8;120(1):94–108. doi: 10.1002/cpt.70311 (PMC13264465; doi:10.1002/cpt.70311)
Supplement: Supplementary file 1 — Figure S1. [file CPT-120-94-s002.docx]

**Figure S1**

**Identification of studies via databases and registers**

Records removed *before screening*:

Duplicate records removed (n = 57)

Records identified from ClinicalTrials.gov:

“DMT” search (n =136)

“Ayahuasca” search (n = 9)

“N,N-Dimethyltryptamine” search (n = 51)

**Identification**

Records excluded

Did not refer to DMT (n = 108)

Observational trial (n = 5)

**Screening**

Records screened

(n = 139)

Records included in review

(n = 26)

Published studies of included trials

(n = 14)

**Included**

**Figure S1. PRISMA 2020 flow diagram for identification, screening, and inclusion of registered clinical trials.** A systematic search of ClinicalTrials.gov was conducted on July 14, 2025 using three expert search queries (“DMT”, “Ayahuasca”, and “N,N-dimethyltryptamine”), yielding 196 records in total. After deduplication by NCT identifier, 57 duplicate records were removed. The remaining records were screened manually for relevance to ayahuasca or N,N-DMT as the investigated intervention. Records in which “DMT” referred to unrelated acronyms, trials involving 5-MeO-DMT, and records retrieved due to psilocybin-related nomenclature overlap were excluded. In addition, observational studies were excluded to restrict the sample to interventional clinical trials. A total of 26 unique interventional registered trials were included in the final review. Published studies associated with included trials were identified through PubMed targeted searches.
