# Supplementary material for: Registered Clinical Trials of Ayahuasca and DMT: A Scoping Review
Source: Clin Pharmacol Ther. 2026 May 8;120(1):94–108. doi: 10.1002/cpt.70311 (PMC13264465; doi:10.1002/cpt.70311)
Supplement: Supplementary file 2 — Figure S2. [file CPT-120-94-s004.docx]

**Figure S2**


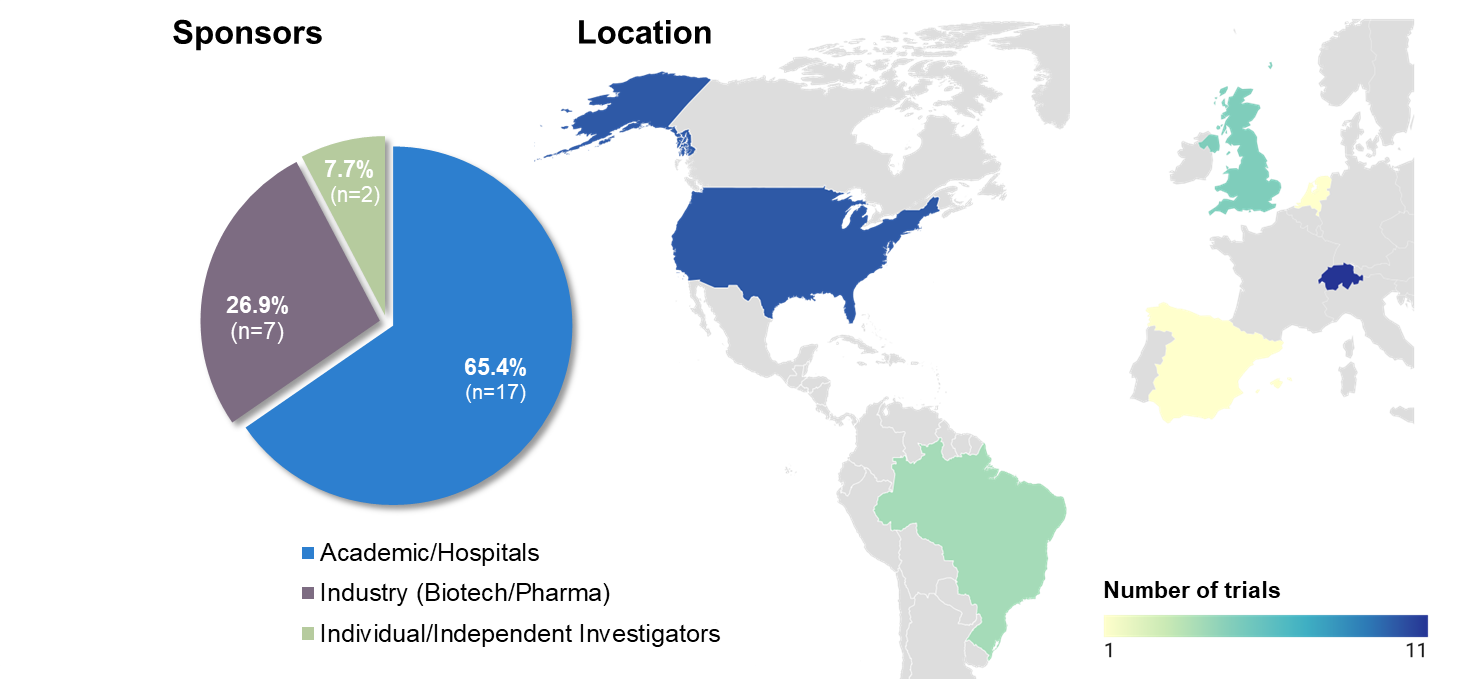


**Figure S2. Sponsor type and geographic distribution of registered ayahuasca and DMT interventional trials. Left panel:** Sponsor category for each trial, classified from the ClinicalTrials.gov sponsor entity as Academic/Hospital, Industry (Biotech/Pharma), or Individual/Independent investigator. Percentages are calculated relative to the 26 included trials. **Right panels:** Country-level distribution of trial locations extracted from ClinicalTrials.gov. Choropleth shading indicates the **number of trials with at least one listed study site** in each country (darker color denotes more trials; numeric range shown in the scale bar).
