# Supplementary material for: Registered Clinical Trials of Ayahuasca and DMT: A Scoping Review
Source: Clin Pharmacol Ther. 2026 May 8;120(1):94–108. doi: 10.1002/cpt.70311 (PMC13264465; doi:10.1002/cpt.70311)
Supplement: Supplementary file 3 — Table S1. [file CPT-120-94-s003.docx]

| Criterion | % Trials | Trial IDs |
| --- | --- | --- |
| Adults who were either psychiatrically healthy or judged cognitively capable of providing informed consent | 88.46 | NCT04711915*; NCT04716335; NCT05901012; NCT06927076*; NCT04353024; NCT05829603; NCT05695495; NCT02033707; NCT05780216; NCT06899334; NCT06070649*; NCT06252506; NCT06671977*; NCT05559931; NCT06180759; NCT06051721*; NCT05384678; NCT04673383*; NCT05553691*; NCT06772753; NCT05644093; NCT02914769*; NCT05894902 |
| Little or no previous experience with psychedelic substances | 15.38 | NCT04716335; NCT05780216; NCT04673383; NCT05644093**** |
| Healthy body mass index (BMI) | 50.00 | NCT04716335; NCT04353024; NCT05695495; NCT05780216; NCT06899334; NCT06252506; NCT06671977; NCT05559931; NCT06180759; NCT06051721; NCT05384678; NCT06772753; NCT05644093 |
| Abstinence from caffeine, alcohol, cigarettes, or psychoactive substances | 69.23 | NCT04716335; NCT06927076; NCT04353024**; NCT05829603; NCT05695495**; NCT02033707#; NCT05780216; NCT06899334; NCT06252506; NCT06671977; NCT05559931; NCT06180759; NCT06051721; NCT05384678**; NCT04673383; NCT06772753#; NCT05644093; NCT05894902 |
| Good knowledge of the local language | 42.31 | NCT04716335; NCT06927076; NCT04353024; NCT05829603; NCT05695495; NCT05780216; NCT06899334; NCT06252506; NCT06671977; NCT06180759; NCT05384678 |
| No driving or operating heavy machinery on the treatment day | 26.92 | NCT06927076; NCT04353024; NCT05695495; NCT06899334; NCT06671977; NCT06180759; NCT05384678 |
| Use of contraceptive measures throughout the study | 50.00 | NCT06927076; NCT04353024; NCT05829603; NCT05695495; NCT05780216; NCT06899334; NCT06671977; NCT05559931; NCT06180759; NCT05384678; NCT04673383; NCT05553691; NCT05644093 |
| Prior experience with DMT/psychedelic substances | 26.92 | NCT05901012; NCT05573568; NCT05829603; NCT02033707; NCT06252506; NCT05644093****; NCT05894902 |
| Vaccination against COVID-19 | 3.85 | NCT05573568 |
| Diagnosis of depressive disorders | 23.08 | NCT04711915; NCT06927076; NCT06094907***; NCT06671977; NCT05553691; NCT02914769¤¤¤ |
| Being right-handed | 3.85 | NCT05901012 |
| No use or stable dose of antidepressants/anxiolytic medications for at least 4 weeks | 19.23 | NCT06927076; NCT06671977¤; NCT06051721; NCT04673383##; NCT05553691¤¤ |
| High school level of education | 3.85 | NCT02033707 |
| Self-reported interest in psychedelic drugs and altered states of consciousness | 3.85 | NCT02033707 |
| Experience in Buddhist meditation | 3.85 | NCT05780216 |
| Diagnosis of alcohol use disorder | 3.85 | NCT06070649 |
| Unsatisfactory response to at least one adequate antidepressant/ anxiolytic medication trial | 11.54 | NCT06671977; NCT06051721; NCT02914769 |
| Negative urine drug screen | 7.69 | NCT06671977*; NCT05644093 |
| Diagnosis of GAD | 3.85 | NCT06051721 |
| No monoamine oxidase-inhibitor class antidepressants for at least 3 months | 3.85 | NCT05553691 |
| No recent psychedelic drug use | 11.54 | NCT05553691; NCT06252506; NCT05644093**** |
| Willing to be contacted by email and video call, and have online access | 15.38 | NCT05553691; NCT04673383; NCT05829603###; NCT05644093 |
| Recent loss of a first-degree relative | 3.85 | NCT06150859 |
| Moderate to high grief symptoms | 3.85 | NCT06150859 |

**Table S1. Inclusion criteria for registered clinical trials of DMT and ayahuasca.** Each row lists an inclusion criterion and the associated registered trial identifiers (NCT IDs). Percentages are calculated as the number of trials listing the criterion divided by the total number of unique trials. Symbols appended to NCT IDs denote trial-specific clarifications as follows: * trial recruits either participants with psychiatric disorders or mixed cohorts; ** abstain from xanthine-containing beverages starting the evening before each study session and during the session; *** participants are in current treatment for depression with partial response; # standardize nicotine/caffeine intake on dosing days to match the participant’s usual morning intake (or abstain if none), refrain for one week before each session from nonprescription medications, supplements, or herbal products unless investigator-approved, and avoid PRN prescription medications on session mornings; ¤ participants must be engaged in clinician-led depression treatment (or initiate it if not engaged at screening) and continue throughout the study, consent to investigator contact with the primary mental health provider, have no current DSM-5 psychiatric disorder except nicotine/caffeine use disorders, and have no lifetime psychiatric medication use >3 months (used as a proxy for psychiatric disorders), with non–use-disorder alcohol/street drug use evaluated case-by-case; ## participants must be off antidepressants or willing to discontinue with adequate washout before and during the study, and must have had no psychedelic drug use in the 6 months prior to dosing; ¤¤ participants must have previously tried ≥1 approved depression treatment, with test cohort on a stable single SSRI (no other psychiatric medications) for ≥6 weeks prior to screening without planned changes, and control cohort off antidepressants for 6 months prior to dosing; ### additional criterion requiring possession of a smartphone capable of running the latest TeleWear and Withings® Health Mate applications; **** two-part design in which Part A allows prior psychedelic experience but requires no psychedelic use within 6 weeks prior to dosing, whereas Part B requires little/no psychedelic experience and no psychedelic use within 6 months prior to dosing; ¤¤¤ current depressive episode defined by HAM-D ≥ 17.
