# Supplementary material for: Registered Clinical Trials of Ayahuasca and DMT: A Scoping Review
Source: Clin Pharmacol Ther. 2026 May 8;120(1):94–108. doi: 10.1002/cpt.70311 (PMC13264465; doi:10.1002/cpt.70311)
Supplement: Supplementary file 4 — Table S2. [file CPT-120-94-s006.docx]

| Criterion | % Trials | Trial IDs |
| --- | --- | --- |
| Previous adverse response to a psychedelic drug | 50.00 | NCT04716335; NCT05901012; NCT05573568; NCT05829603; NCT06094907; NCT05780216&; NCT06252506; NCT06671977; NCT05559931; NCT06051721; NCT05553691; NCT04673383; NCT05644093 |
| Participation in another clinical trial | 57.69 | NCT04716335; NCT04353024; NCT05829603; NCT05695495; NCT05780216; NCT06899334; NCT06252506; NCT06671977; NCT05559931; NCT06180759; NCT06051721; NCT05384678; NCT04673383; NCT06772753; NCT05644093 |
| Current or recent use of psychotropic treatments | 61.54 | NCT04716335; NCT06927076; NCT04353024; NCT05829603; NCT05695495; NCT02033707; NCT05780216; NCT06899334; NCT06252506; NCT06671977; NCT06180759; NCT06051721###; NCT05384678; NCT05553691###; NCT05894902; NCT04673383 |
| Pregnancy or breastfeeding | 84.62 | NCT05901012; NCT06927076; NCT04353024; NCT05573568; NCT05829603; NCT05695495; NCT06094907; NCT02033707; NCT05780216; NCT06899334; NCT06671977; NCT05559931; NCT06180759; NCT06051721; NCT05384678; NCT04673383; NCT05553691; NCT06772753; NCT05644093; NCT02914769; NCT06150859; NCT05894902 |
| Severe obesity | 11.54 | NCT05901012; NCT05573568; NCT06094907 |
| Diagnosis or family suspicion of genetic monoamine oxidase deficiency | 11.54 | NCT05901012; NCT05573568; NCT06094907 |
| Chronic or recent use of psychedelics | 42.31 | NCT06927076; NCT04353024; NCT05695495; NCT06899334; NCT06671977; NCT05559931; NCT06180759; NCT05384678; NCT05553691; NCT04673383##; NCT02914769 |
| Ongoing 5-HT2R antagonist, MAOI, CYP2C9 or CYP3A4 inhibitor treatments | 42.31 | NCT06927076; NCT05829603; NCT02033707; NCT05780216; NCT06252506; NCT06671977; NCT05559931; NCT06051721; NCT04673383; NCT06772753; NCT05894902 |
| High risk of adverse emotional or behavioral reaction | 15.38 | NCT05829603; NCT05780216; NCT06252506; NCT06772753 |
| Schizoaffective disorders | 80.77 | NCT05901012; NCT06927076; NCT04353024; NCT05573568; NCT05829603; NCT05695495; NCT06094907; NCT02033707; NCT05780216; NCT06899334; NCT06252506; NCT06671977; NCT05559931; NCT06180759; NCT06051721; NCT05384678; NCT05553691; NCT06772753****; NCT05644093; NCT02914769; NCT06150859 |
| Depressive disorders | 19.23 | NCT05829603; NCT02033707; NCT05780216; NCT06252506; NCT06150859 |
| Bipolar affective disorder | 80.77 | NCT05901012; NCT06927076; NCT04353024; NCT05573568; NCT05829603; NCT05695495; NCT06094907; NCT02033707; NCT05780216; NCT06899334; NCT06252506; NCT06671977; NCT05559931; NCT06180759; NCT06051721; NCT05384678; NCT05553691; NCT06772753; NCT05644093; NCT02914769; NCT06150859 |
| Substance-related and addictive disorders | 65.38 | NCT05901012#; NCT06927076¤; NCT05573568#; NCT05829603; NCT06094907#; NCT05780216; NCT06252506; NCT06671977¤####; NCT05559931; NCT06051721; NCT04673383; NCT05553691; NCT06772753#; NCT05644093##; NCT02914769; NCT06150859¤; NCT05894902 |
| Nicotine addiction | 11.54 | NCT04716335; NCT05829603; NCT05780216 |
| Acute or subacute risk of suicide | 42.31 | NCT05901012; NCT06927076; NCT05573568; NCT06094907; NCT06671977; NCT05559931; NCT06051721; NCT05553691; NCT06772753; NCT05644093; NCT02914769 |
| Present or antecedent psychiatric disorders | 38.46 | NCT04716335; NCT04353024; NCT05695495; NCT06899334; NCT05559931; NCT06180759; NCT05384678; NCT05644093; NCT05894902; NCT06671977 |
| Psychiatric disorders other than depressive disorders/GAD | 19.23 | NCT04711915*; NCT06671977; NCT06051721; NCT04673383; NCT05553691 |
| Dissociative disorders | 26.92 | NCT05901012; NCT05573568; NCT06094907; NCT02033707; NCT05780216; NCT06252506; NCT06150859 |
| Anxiety disorders | 26.92 | NCT05829603; NCT05695495; NCT02033707; NCT05780216; NCT06252506; NCT05384678; NCT06150859 |
| Borderline personality disorder | 7.69 | NCT06927076; NCT06051721 |
| Ongoing post-traumatic stress disorder | 7.69 | NCT06927076; NCT06150859 |
| Postpartum depression | 7.69 | NCT06927076; NCT06150859 |
| Cardiovascular disorders | 84.62 | NCT04716335; NCT05901012; NCT04353024; NCT05573568; NCT05829603; NCT05695495; NCT06094907; NCT02033707; NCT05780216; NCT06899334; NCT06252506; NCT06671977; NCT05559931; NCT06180759; NCT06051721; NCT05384678; NCT04673383; NCT05553691; NCT06772753; NCT05644093; NCT06150859; NCT05894902 |
| Pulmonary disorders | 34.62 | NCT05573568; NCT05901012; NCT05829603; NCT06094907; NCT05780216; NCT06252506; NCT05559931; NCT06051721; NCT05644093 |
| Renal disorders | 46.15 | NCT04716335; NCT05901012; NCT05573568; NCT05829603; NCT06094907; NCT05780216; NCT06252506; NCT06671977; NCT05559931; NCT06051721; NCT05644093; NCT05894902 |
| Endocrine disorders | 11.54 | NCT05559931; NCT06051721; NCT05644093 |
| Hepatic disorders | 46.15 | NCT04716335; NCT05901012; NCT05573568; NCT05829603; NCT06094907; NCT05780216; NCT06252506; NCT06671977; NCT05559931; NCT06051721; NCT05644093; NCT05894902 |
| Gastrointestinal disorders | 3.85 | NCT06051721 |
| Recent cardiac or brain surgery | 15.38 | NCT04716335; NCT05829603; NCT05780216; NCT06252506 |
| Acute airway infection or flu | 11.54 | NCT05901012; NCT05573568; NCT06094907 |
| Recent COVID-19 exposure | 7.69 | NCT05901012; NCT06094907 |
| Neurological and cerebrovascular disease | 53.85 | NCT04716335; NCT05901012; NCT05573568; NCT05829603; NCT06094907; NCT02033707; NCT05780216; NCT06252506; NCT06671977; NCT05559931; NCT06051721; NCT06772753; NCT05644093; NCT05894902 |
| General chronic or acute medical condition | 73.08 | NCT04711915; NCT04716335; NCT04353024; NCT05829603; NCT05695495; NCT05780216; NCT06899334; NCT06070649; NCT06252506; NCT06671977; NCT05559931; NCT06180759; NCT06051721; NCT05384678; NCT04673383; NCT05553691; NCT05644093; NCT02914769; NCT05894902 |
| Cancer | 15.38 | NCT05829603; NCT05780216; NCT06252506; NCT06051721 |
| Metabolic disorders | 34.62 | NCT02033707; NCT06252506; NCT05901012; NCT05573568; NCT06094907; NCT06671977; NCT05559931; NCT06772753; NCT05644093 |
| Unhealthy BMI | 7.69 | NCT02033707; NCT05894902 |
| Anorexia nervosa or bulimia nervosa | 7.69 | NCT02033707; NCT06150859 |
| Obsessive-Compulsive Disorder | 3.85 | NCT02033707 |
| MRI/PET exclusion criteria | 11.54 | NCT05780216; NCT06252506; NCT05559931 |
| Left-handedness | 7.69 | NCT05780216; NCT06252506 |
| Frequent tobacco smoking | 26.92 | NCT06899334; NCT04353024; NCT05695495; NCT05559931***; NCT06180759; NCT05384678; NCT04673383 |
| Excessive consumption of alcohol | 34.62 | NCT06899334; NCT04353024; NCT05695495; NCT06671977; NCT05559931; NCT06180759; NCT05384678; NCT05644093; NCT06150859 |
| Serious abnormalities in ECG or blood count/chemistry | 34.62 | NCT05829603; NCT05780216; NCT06252506; NCT05559931; NCT06051721; NCT06772753; NCT05644093; NCT02914769; NCT05894902 |
| Clinically significant aggressive behavior | 3.85 | NCT06671977 |
| Cognitive dysfunction | 3.85 | NCT06671977 |
| Excessive consumption of caffeine | 7.69 | NCT05559931; NCT06051721 |
| Insufficient knowledge of the local language | 3.85 | NCT06051721 |
| Difficulty in establishing rapport | 19.23 | NCT06051721; NCT05559931; NCT02033707; NCT04673383; NCT05644093 |
| Recent or planned donation of blood or plasma | 11.54 | NCT06051721; NCT05559931; NCT04673383; NCT05644093 |
| Planned sperm donation during the study | 3.85 | NCT06051721 |
| Positive test for hepatitis B or C, HIV | 11.54 | NCT05559931; NCT04673383; NCT05644093 |
| Allergy/adverse reaction to any medications | 11.54 | NCT06772753; NCT05559931; NCT05644093 |
| Recent use of specific over-the-counter or prescribed medication | 23.08 | NCT04673383; NCT05553691; NCT05559931; NCT06772753; NCT05644093; NCT06150859 |
| Phobia of, or vein unsuitability for, i.v. or blood sampling | 7.69 | NCT05644093; NCT04673383 |
| Receiving psychological or self-help therapy during the study | 3.85 | NCT06150859 |
| Prior relationship to the study or investigators | 3.85 | NCT05559931 |

**Table S2. Exclusion criteria for registered clinical trials of DMT and ayahuasca.** Each row lists an exclusion criterion and the associated NCT IDs; percentages are calculated as the number of trials listing the criterion divided by the total number of unique trials. Symbols appended to NCT IDs denote trial-specific clarifications as follows: * healthy control cohort required medical and psychiatric health, while the depression cohort required an MDD diagnosis and otherwise medical health; # exclusion applies except tobacco; ¤ exclusion applies except nicotine; & exclusion includes previous significant adverse response to a hallucinogenic drug or to a mindfulness intervention (e.g., meditation retreat); *** criterion explicitly includes nicotine use; % excessive caffeine/(methyl)xanthine intake as determined by investigator judgment; ### antidepressants, anxiolytics, and sedatives/hypnotics are permitted; ## criterion includes cannabis use within 24 h prior to each study visit; **** psychotic-spectrum exclusion does not include Substance/Medication-Induced Psychotic Disorder or Psychotic Disorder Due to Another Medical Condition; #### exclusion applies except caffeine and nicotine.
