# Supplementary material for: Registered Clinical Trials of Ayahuasca and DMT: A Scoping Review
Source: Clin Pharmacol Ther. 2026 May 8;120(1):94–108. doi: 10.1002/cpt.70311 (PMC13264465; doi:10.1002/cpt.70311)
Supplement: Supplementary file 6 — File S2. [file CPT-120-94-s007.docx]

**Primary outcome measures**

- Change in behavioral outcome measures
  - Social Value Orientation - SVO, Charity Donation Frank Task
  - Visual Oddball, Karaoke Task
- Changes in EEG
  - Lagged Phase Synchronicity
  - Resting State
  - Change in Pharmacological-EEG
- Changes in anxiety symptoms
  - Hamilton Anxiety Rating Scale (HAM-A)
  - Acute Anxiety Response Questionnaire
- Change in respiratory rate
- Change in oxygen saturation
- Subjective effect ratings
  - Over time
  - Subjective effects
- Changes in depressive symptoms
  - Change in MADRS scores
  - Self-assessed depressive symptoms
  - Hamilton Depression Rating Scale (HAM-D) Effect Seven Days After Dosing (D7)
  - Long term changes
- Altered state of consciousness profile (ASC, 5D-ASC)
- Overall tolerability and safety
  - Assessed by the VAS
  - Safety and tolerability in healthy volunteers
  - Tolerability of Overt Adverse Effects
  - Safety and Tolerability: proportion of subjects with at least one adverse event (AE)
  - Safety of Physiological indices
  - Safety and tolerability of DMT in women and men with AUD
  - Safety and Tolerability: Proportion of subjects with abnormal vital signs
  - Safety and Tolerability: Proportion of subjects with abnormal ECG readings
  - Safety and Tolerability: Proportion of subjects with abnormal physical examination findings
  - Safety and Tolerability: percentage of subjects with abnormal haematology, clinical chemistry, coagulation, and urinalysis values
  - Safety and Tolerability: percentage of subjects with local reactions at the injection site
  - Safety and Tolerability: proportion of subjects with abnormal findings on the Columbia-Suicide Severity Ratings Scale (C-SSRS)
  - Safety and Tolerability: proportion of subjects with occurrence of psychotic symptoms (BPRS)
  - Safety and Tolerability: proportion of subjects with occurrence of central 5-HT toxicity
  - Safety and Tolerability: proportion of subjects with at least one adverse event (AE)
  - Safety & tolerability: Adverse Events
- Incidence of treatment-emergent adverse events
  - Adverse clinical and psychiatry symptoms assessed by qualitative medical/clinical-psychiatry evaluation
- Pharmacokinetic parameter "Cmax"
- Pharmacokinetic parameter "Area under the curve (AUC)"
- Pharmacokinetic parameter "T1/2"
- Genotyping
- Subjective drug valuation and Preference
  - Rating of "Drug Liking" on the End of Day Questionnaire
  - Reinforcement and Abuse Liability
- Functional brain connectivity changes (rs-fMRI)
- Effects on alcohol consumption
- Change from baseline in cerebral metabolic rate for glucose (CMRglc)
- Electrophysiological
- Psychotomimetic effects
- Percentage of subjects with local reactions at the injection site
- Occurrence of psychotic symptoms (BPRS)
- Occurrence of central 5-HT toxicity
- Changes in grief symptom severity
- Pain assessment (NRS) - Numeric rating scale (NRS) scores (0 - 10) - Difference of the cumulative NRS scores (area under the effect curves (AUECs)) between the DMT, ketamine (active-control) and placebo condition. NRS 0 represents "no pain at all" whereas 10 represents "the worst pain ever possible".
- Mystical Experience Questionnaire (MEQ-30) - The MEQ is a 30-item self-report instrument intended to assess psychedelic-specific acute subjective effects of psychoactive drugs. At the end of each experimental session, participants will be instructed to rate each of the items of the MEQ relative to the experiences that the participant encountered during the course of the drug administration session. The total score on all items is then calculated as the primary outcome measure for each experimental session. Scores range from 0 to 150 on this measure with higher scores reflecting more mystical experience
- Suicidal ideation
  - Beck scale for suicidal ideation (BSS)
  - Safety and Tolerability: proportion of subjects with abnormal findings on the Columbia-Suicide Severity Ratings Scale (C-SSRS)
- Electrocardiogram (ECG)
  - QT interval (12-lead Electrocardiogram [ECG])
  - Proportion of subjects with abnormal ECG readings
- Physical exam
  - Safety and Tolerability: Proportion of subjects with abnormal physical examination findings
- Blood and urine analysis
  - Safety and Tolerability: percentage of subjects with abnormal haematology, clinical chemistry, coagulation, and urinalysis values
  - Blood count (Lab biochemistry)
  - Clinical chemistry (Lab biochemistry)
  - Blood coagulation (Lab biochemistry)
  - Clinical laboratory assessments
- Vital signs - blood pressure, heart rate, or temperature
  - Pulse oximetry
